# Social Inequalities in Secondhand Smoke Among Japanese Non-smokers: A Cross-Sectional Study

**DOI:** 10.2188/jea.JE20160184

**Published:** 2018-03-05

**Authors:** Yusuke Matsuyama, Jun Aida, Toru Tsuboya, Shihoko Koyama, Yukihiro Sato, Atsushi Hozawa, Ken Osaka

**Affiliations:** 1 Department of International and Community Oral Health, Tohoku University Graduate School of Dentistry, Sendai, Japan; 2 Department of Preventive Medicine and Epidemiology, Tohoku Medical Megabank Organization, Tohoku University, Sendai, Japan

**Keywords:** tobacco, social inequalities, secondhand smoke

## Abstract

**Background:**

Secondhand smoke (SHS) causes many deaths. Inequalities in SHS have been reported in several countries; however, the evidence in Asian countries is scarce. We aimed to investigate the association between socioeconomic status (SES) and SHS at home and the workplace/school among non-smoking Japanese adults.

**Methods:**

Cross-sectional data from the Miyagi Prefectural Health Survey 2014 were analyzed. Self-reported questionnaires were randomly distributed to residents ≥20 years of age and 2,443 (92.8%) responded. The data of the 1,738 and 1,003 respondents were included to the analyses for SHS in the past month at home and at the workplace/school, respectively. Ordered logistic regression models considering possible confounders, including knowledge of the adverse health effects of tobacco, were applied.

**Results:**

The prevalence of SHS at home and the workplace/school was 19.0% and 39.0%, respectively. Compared with ≥13 years of education, odds ratios (ORs) and 95% confidence intervals (CIs) for SHS at home were 1.94 (95% CI, 1.42–2.64) for 10–12 years and 3.00 (95% CI, 1.95–4.60) for ≤9 years; those for SHS at the workplace/school were 1.80 (95% CI, 1.36–2.39) and 3.82 (95% CI, 2.29–6.36), respectively. Knowledge of the adverse health effects of tobacco was significantly associated with lower SHS at home (OR 0.95; 95% CI, 0.91–0.98) but it was not associated with SHS at the workplace/school (OR 1.02; 95% CI, 0.98–1.06).

**Conclusions:**

Social inequalities in SHS existed among Japanese non-smoking adults. Knowledge about tobacco was negatively associated with SHS at home but not at workplace/school.

## INTRODUCTION

Secondhand smoke (SHS) seriously burdens population health and society. Worldwide, an estimated 603,000 deaths, approximately 1.0% of worldwide mortality, were attributed to SHS^1^; and loss of disability-adjusted life-years (DALYs) due to SHS reached 10.9 million years, which is about 0.7% of all DALYs burdened by diseases.^1^ Previous studies showed robust evidence of the causality between SHS and various diseases, such as coronary artery disease, lung cancer, and stroke.^2^ Any level of SHS should be reduced because mechanisms of SHS and respiratory diseases suggest that there is no safe level of exposure to SHS.^3^

The economic costs of SHS are also enormous. In the United States, yearly productivity losses following SHS-related death are estimated at $6.6 billion.^4^ It is estimated that healthcare costs of $49 million a year can be saved if all workplaces were smoke free.^5^ In the United Kingdom, nineteen percent of total expenditures on childhood respiratory conditions are due to SHS, and $600 million for children and $19 million for adults is spent on conditions related to SHS.^6^ In China, the largest consumer country of tobacco in the world, total SHS-related healthcare costs are estimated at $1.2 billion in rural areas alone.^7^

In addition to these adverse effects of SHS, social inequalities in SHS are another public health problem. Several European and American studies have shown that inequalities in SHS still exist.^8^^–^^10^ However, in Asia, where cigarettes are most consumed in the world,^11^ evidence of inequalities in SHS is scarce: eight studies^12^^–^^19^ have reported social inequalities in SHS. Especially in Japan, to the best of our knowledge, only one study has reported inequalities in SHS: people with lower educational attainment had higher prevalence of SHS at home and at the workplace.^19^ Public places in Japan are not protected by strict smoke-free legislation,^20^ even though cigarette consumption in Japan and health risks due to cigarettes are high.^11^ Therefore, we aimed to investigate the association between socioeconomic status (SES) and SHS among non-smoking Japanese adults.

## METHODS

### Study participants

A cross-sectional study with secondary analyses using data from the Miyagi Prefectural Health Survey 2014 was conducted. In October and November 2014, 2,632 people were randomly selected from residents aged ≥20 years in Miyagi Prefecture, Japan and were sent self-reported questionnaires. Among the 2,632 residents who received the questionnaires, 2,443 (92.8%) responded to the survey. The distribution of self-reported smoking status obtained from the following single question: “*Have you ever smoked?*” among the 2,443 respondents was 565 current smokers (23.1%), 528 former smokers (21.6%), 1,254 never smokers (51.3%), and 96 with missing information on their smoking status (3.9%). The data of non-smokers (never and former smokers) was used in the present study. Ethical approval for this secondary analysis was obtained from the Ethics Committee of Tohoku University.

### Dependent variables

Frequencies of SHS at home and at the workplace/school in the past month were obtained from two questions: Q1) *“During the past month, were you exposed to secondhand smoke at home?”* and Q2) *“During the past month, were you exposed to second hand smoke at the workplace/school?”* The possible answers were *“none,” “once a month,” “once a week,” “sometimes a week,” “almost every day,” and “don’t go to such places/don’t know”*. Respondents who answered *“don’t go to such places/don’t know”* were excluded from the analyses of the present study. *“Once a month”* and *“once a week”* were categorized as “once a week or less”. Therefore, we used two dependent variables, SHS at home and SHS at the workplace/school, which had four ordered categories of “none,” “once a week or less,” “sometimes *a week*,” and “almost every day”. Respondents with no occupation and housekeepers were excluded from the analyses for SHS at the workplace/school.

### Predictor variables

Our main predictors were years of education and equalized household annual income. These variables are good indicators of health inequalities and have been used in various studies.^21^ Years of education was asked using the following single question: “*How many years of education do you have?*” The answer was chosen from three categories: ≤9 years, 10–12 years, and ≥13 years. Household annual income was asked using the following single question: “*How much was your household annual income in the last year?*” The answer was chosen from three categories: <2.0 million JPY, 2.0–5.9 million JPY, ≥6.0 million JPY, and don’t know (approximately 100 JPY = 1 USD). Answer of “don’t know” was treated as missing information. Equalized household annual income was calculated by dividing the household annual income by the square root of the number of people in household, and used as tertile categories (low: ≤2 million JPY, middle: 2.1–2.9 million JPY, and high: ≥3.0 million JPY).

### Covariates

To consider individual knowledge of tobacco’s adverse effects on health, the participants were asked their knowledge of tobacco’s effect on 11 diseases via the following question: *“Do you think smoking causes [lung cancer, larynx cancer, asthma, bronchitis, chronic obstructive pulmonary disease, heart disease, stroke, gastric ulceration, adverse effects on unborn children, periodontal disease, and hypertension]?”* The answers choices were *“caused by smoking,” “don’t know the association,” “not caused by smoking,”* and *“don’t know the disease.”* We categorized *“caused by smoking”* as “1” and the other answers as “0.” Scores for knowledge of the adverse health effects of tobacco were determined by summing these values to create a continuous variable ranging from 0–11. Age (20–39 years, 40–59 years, or 60 years-old or more), sex (male or female), number of people in household (continuous variables of 1, 2, 3, 4, or 5 or more), and self-reported smoking status (never smoker, or former smoker) were also adjusted.

### Statistical analyses

Ordered logistic regression models were fitted to estimate odds ratios (ORs) and 95% confidence intervals (CIs) of education and income for SHS at home and at the workplace/school, respectively. The Brant test with complete case data showed that proportional odds assumption was not violated (*P* = 0.229 for SHS at home; *P* = 0.634 for SHS at the workplace/school), so ordered logistic regression models were appropriate.^22^ Four models were estimated for each outcome: income and education were separately included, and age and sex were adjusted (models 1 & 2); income and education were simultaneously included, and age and sex were adjusted (model 3); and finally, the number of people in household, smoking status, and knowledge of tobacco’s adverse health effects were added to model 3 (model 4). Then, complete case analyses and analyses with multiple imputed datasets aiming to consider bias due to missing information were conducted. In the multiple imputation procedure, 20 multiple imputed datasets were made. Missing at random was assumed, and the multivariate normal imputation method was applied. The estimated parameters were then combined using Rubin’s combination methods.^23^^,^^24^ All analyses were performed using Stata software (version 14.0; Stata Corp LP, College Station, TX, USA) at a significance level of 0.05.

## RESULTS

**Figure 1.**
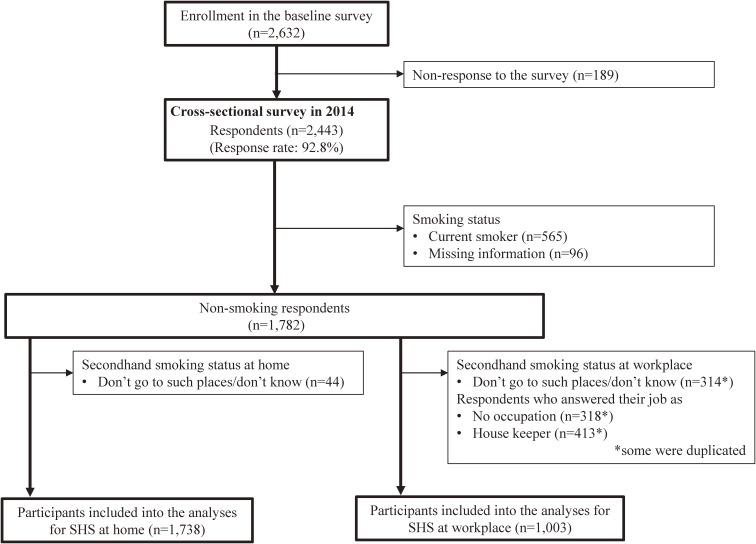
Flowchart of study participants. SHS, secondhand smoke.

Figure 1 shows the flowchart of the study participants. As SHS at home and SHS at the workplace/school were separately analyzed, the numbers of analytical participants were different according to the dependent variable: 1,738 for SHS at home and 1,003 for SHS at the workplace/school.

Table 1 and Table 2 show the demographic characteristics of the participants and their SHS status. Among the participants, 19.0% were exposed to SHS at home, while 39.0% were exposed to SHS at the workplace/school. The characteristics of participants exposed to SHS at home were: younger, women, those with lower educational attainment, lower income, living with a larger number of people in household, never smokers, and those with poor knowledge of tobacco’s adverse health effects (Table 1). The characteristics of participants exposed to SHS at the workplace/school were: younger, men, those with lower educational attainment, and former smokers (Table 2).

**Table 1.** Characteristics of the analytical participants for secondhand smoke at home (*n* = 1,738)

	Total	Second hand smoke at home										

		None		Once a week or less		Several times a week		Almost everyday		Missing		*P*-value^a^
	*n*	*n*	%	*n*	%	*n*	%	*n*	%	*n*	%	
Age, years												
20–39	350	238	68.0	25	7.1	21	6.0	51	14.6	15	4.3	<0.001
40–59	620	455	73.4	22	3.5	21	3.4	70	11.3	52	8.4	
≥60	768	459	59.8	42	5.5	24	3.1	54	7.0	189	24.6	
Sex												
Men	682	496	72.7	29	4.3	12	1.8	20	2.9	125	18.3	<0.001
Women	1,056	656	62.1	60	5.7	54	5.1	155	14.7	131	12.4	
Education, years												
≤9	296	139	47.0	19	6.4	12	4.1	28	9.5	98	33.1	<0.001
10–12	806	519	64.4	44	5.5	31	3.8	99	12.3	113	14.0	
≥13	627	492	78.5	26	4.1	23	3.7	47	7.5	39	6.2	
Missing	9	2	22.2	0	0.0	0	0.0	1	11.1	6	66.7	
Income												
Low	700	418	59.7	44	6.3	30	4.3	85	12.1	123	17.6	<0.001
Middle	575	419	72.9	27	4.7	14	2.4	47	8.2	68	11.8	
High	334	258	77.2	11	3.3	10	3.0	25	7.5	30	9.0	
Missing	129	57	44.2	7	5.4	12	9.3	18	14.0	35	27.1	
Smoking												
Former smoker	515	356	69.1	22	4.3	16	3.1	30	5.8	91	17.7	<0.001
Never smoker	1,223	796	65.1	67	5.5	50	4.1	145	11.9	165	13.5	
Total	1,738	1,152	66.3	89	5.1	66	3.8	175	10.1	256	14.7	

	mean (SD)	mean (SD)		mean (SD)		mean (SD)		mean (SD)		mean (SD)		*P*-value^b^

Number of people in household^c^	3.3 (1.3)	3.2 (1.2)		3.6 (1.3)		3.5 (1.4)		3.6 (1.1)		2.9 (1.3)		<0.001
Knowledge of the adverse health effects of tobacco^d^	7.2 (3.4)	7.4 (3.3)		6.7 (3.4)		6.2 (3.9)		6.5 (3.5)		7.3 (3.6)		0.002

SD, standard deviation.

^a^*P*-value from chi-squared test.

^b^*P*-values from analyses of variance.

^c^Missing information (*n* = 303) were excluded.

^d^Missing information (*n* = 3) were excluded.

**Table 2.** Characteristics of the analytical participants for secondhand smoke at the workplace/school (*n* = 1,003)

	Total	Second hand smoke at workplace/school										

		None		Once a week or less		Several times a week		Almost everyday		Missing		*P*-value^a^
	*n*	*n*	%	*n*	%	*n*	%	*n*	%	*n*	%	
Age, years												
20–39	300	150	50.0	41	13.7	39	13.0	62	20.7	8	2.7	<0.001
40–59	488	267	54.7	57	11.7	53	10.9	72	14.8	39	8.0	
≥60	215	103	47.9	25	11.6	23	10.7	18	8.4	46	21.4	
Sex												
Men	486	213	43.8	64	13.2	63	13.0	94	19.3	52	10.7	<0.001
Women	517	307	59.4	59	11.4	52	10.1	58	11.2	41	7.9	
Education, years												
≤9	80	20	25.0	9	11.3	10	12.5	16	20.0	25	31.3	<0.001
10–12	445	211	47.4	50	11.2	60	13.5	75	16.9	49	11.0	
≥13	476	289	60.7	64	13.4	45	9.5	61	12.8	17	3.6	
Missing	2	0	0.0	0	0.0	0	0.0	0	0.0	2	100.0	
Income												
Low	392	182	46.4	44	11.2	47	12.0	67	17.1	52	13.3	<0.001
Middle	317	171	53.9	42	13.2	33	10.4	51	16.1	20	6.3	
High	241	146	60.6	32	13.3	26	10.8	26	10.8	11	4.6	
Missing	53	21	39.6	5	9.4	9	17.0	8	15.1	10	18.9	
Smoking												
Former smoker	342	159	46.5	50	14.6	37	10.8	60	17.5	36	10.5	<0.001
Never smoker	661	361	54.6	73	11.0	78	11.8	92	13.9	57	8.6	
Total	1,003	520	51.8	123	12.3	115	11.5	152	15.2	93	9.3	

	mean (SD)	mean (SD)		mean (SD)		mean (SD)		mean (SD)		mean (SD)		*P*-value^b^

Number of people in household^c^	3.4 (1.2)	3.4 (1.2)		3.3 (1.1)		3.2 (1.3)		3.6 (1.2)		3.4 (1.2)		0.175
Knowledge of the adverse health effects of tobacco^d^	7.4 (3.3)	7.4 (3.4)		7.3 (3.0)		7.6 (3.3)		7.2 (3.4)		7.5 (3.7)		0.908

SD, standard deviation.

^a^*P*-value from chi-squared test.

^b^*P*-values from analyses of variance.

^c^Missing information (*n* = 114) were excluded.

^d^Missing information (*n* = 2) were excluded.

Table 3 shows the estimated ORs and 95% CIs for SHS at home using multiple imputed datasets. Low educational attainment and low income were significantly associated with SHS at home (models 1–3). After considering all covariates, low educational attainment was significantly associated with SHS at home (model 4: OR 1.94; 95% CI, 1.42–2.64 for 10–12 years of education and OR 3.00; 95% CI, 1.95–4.60 for ≤9 years of education). Income was not significantly associated with SHS at home (model 4: OR 1.04; 95% CI, 0.69–1.55 for middle income and OR 1.44; 95% CI, 0.98–2.11 for low income). Higher knowledge of tobacco’s adverse health effects was significantly associated with low SHS at home (OR 0.95; 95% CI, 0.91–0.98).

**Table 3.** Odds ratios for secondhand smoke at home (*n* = 1,738); multiple imputation was applied

	Model 1		Model 2		Model 3		Model 4	
			
	OR	95% CI	OR	95% CI	OR	95% CI	OR	95% CI
Age, years								
20–39	1.00	ref.	1.00	ref.	1.00	ref.	1.00	ref.
40–59	0.55	0.40, 0.75	0.70	0.52, 0.96	0.59	0.43, 0.81	0.61	0.45, 0.85
≥60	0.45	0.32, 0.64	0.78	0.58, 1.05	0.48	0.34, 0.68	0.51	0.35, 0.73
Sex								
Men	1.00	ref.	1.00	ref.	1.00	ref.	1.00	ref.
Women	2.65	2.01, 3.50	2.71	2.05, 3.58	2.68	2.03, 3.55	2.79	2.03, 3.83
Education, years								
≥13	1.00	ref.			1.00	ref.	1.00	ref.
10–12	2.22	1.65, 2.99			2.03	1.49, 2.76	1.94	1.42, 2.64
≤9	3.68	2.43, 5.56			3.17	2.08, 4.84	3.00	1.95, 4.60
Income								
High			1.00	ref.	1.00	ref.	1.00	ref.
Middle			1.28	0.87, 1.90	1.10	0.74, 1.65	1.04	0.69, 1.55
Low			2.12	1.48, 3.02	1.66	1.15, 2.39	1.44	0.98, 2.11
Number of family members							1.09	0.98, 1.20
Smoking status								
Cessation smoker							1.00	ref.
Never smoker							0.91	0.66, 1.24
Knowledge								
Knowledge of tobacco’s adverse effect							0.95	0.91, 0.98

CI, confidence interval; OR, odds ratio; ref, reference.

Table 4 shows the estimated ORs and 95% CIs for SHS at the workplace/school using multiple imputed datasets. Low educational attainment and low income were significantly associated with SHS at the workplace/school (models 1–3). After considering all covariates, low educational attainment was significantly associated with SHS at the workplace/school (model 4: OR 1.80; 95% CI, 1.36–2.39 for 10–12 years of education and OR 3.82; 95% CI, 2.29–6.36 for ≤9 years of education). Income was significantly associated with SHS at the workplace/school (model 4: OR 1.32 95% CI, 0.93–1.88 for middle income and OR 1.57; 95% CI, 1.09–2.26 for low income). Knowledge of tobacco’s adverse health effects was not significantly associated with SHS at the workplace/school (OR 1.02; 95% CI, 0.98–1.06). The associations were robust when only the data of complete cases were analyzed (results are available upon request).

**Table 4.** Odds ratios for secondhand smoke at the workplace/school (*n* = 1,003); multiple imputation was applied

	Model 1		Model 2		Model 3		Model 4	
			
	OR	95% CI	OR	95% CI	OR	95% CI	OR	95% CI
Age, years								
20–39	1.00	ref.	1.00	ref.	1.00	ref.	1.00	ref.
40–59	0.62	0.47, 0.83	0.74	0.56, 0.98	0.65	0.48, 0.87	0.63	0.47, 0.84
≥60	0.34	0.22, 0.51	0.52	0.36, 0.76	0.34	0.22, 0.52	0.32	0.21, 0.49
Sex								
Men	1.00	ref.	1.00	ref.	1.00	ref.	1.00	ref.
Women	0.46	0.36, 0.60	0.46	0.36, 0.60	0.46	0.35, 0.60	0.46	0.35, 0.62
Education, years								
≥13	1.00	ref.			1.00	ref.	1.00	ref.
10–12	1.92	1.46, 2.52			1.78	1.35, 2.36	1.80	1.36, 2.39
≤9	4.13	2.51, 6.78			3.73	2.25, 6.19	3.82	2.29, 6.36
Income								
High			1.00	ref.	1.00	ref.	1.00	ref.
Middle			1.52	1.08, 2.14	1.29	0.91, 1.83	1.32	0.93, 1.88
Low			1.82	1.30, 2.53	1.43	1.01, 2.01	1.57	1.09, 2.26
Number of family members							0.92	0.82, 1.03
Smoking status								
Cessation smoker							1.00	ref.
Never smoker							0.96	0.72, 1.28
Knowledge								
Knowledge of tobacco’s adverse effect							1.02	0.98, 1.06

CI, confidence interval; OR, odds ratio; ref, reference.

## DISCUSSION

This study investigated the social inequalities in SHS in Japan. The prevalence of exposure to SHS at least once a month was 19.0% at home and 39.0% at the workplace/school. These prevalences are similar to those reported in a national survey in Japan.^25^ People with lower educational attainment had significantly higher odds of being exposed to SHS at home and at the workplace/school. As to SHS at the workplace/school, lower income was significantly associated with higher SHS. Knowledge of tobacco’s adverse health effects seemed to reduce SHS at home but not at the workplace/school.

Some previous studies have reported social inequalities in SHS. In Japan, at least one study has been reported. In the study, one in four Japanese adults was exposed to SHS daily. The prevalence of SHS in the study was higher among people with lower education.^19^ In other Asian countries, it is reported that lower parental education level was associated with higher SHS at home among students.^18^ Lower education and lower income were associated with higher SHS both at home and at the workplace, and the gradient was clearer in education.^14^ In European countries, Nazar et al reported that 11 of 15 low- or middle-income countries showed social inequalities in SHS at home or the workplace. In that study, the education-related social inequalities were more obvious than the income-related social inequalities.^26^ The present study showed similar results to these previous studies.

Interestingly, the present study showed that knowledge of tobacco’s adverse health effects was significantly associated with low SHS at home; however, it was not associated with SHS at the workplace/school. This suggests that people cannot reduce their exposure to SHS at the workplace/school even if they are aware of the dangers of tobacco. Therefore, education about tobacco’s adverse health effects alone is insufficient to reduce SHS. Previous studies have shown that a comprehensive, nationwide approach, such as price/tax increases, is one possible intervention that can reduce the social inequalities in SHS.^27^

Previous studies have suggested links between SES and SHS. The indoor smoking ban at home is less common among households with low SES.^28^ Therefore, smokers with low SES tend to smoke not only outside but also in their home, which may contribute to inequalities in SHS at home. Our results, which demonstrate the negative association between knowledge of tobacco’s adverse health effects and SHS at home, support this interpretation. Education targeting the adverse health effects of tobacco use would help to reduce SHS. In addition, smoking inside vehicles, in the presence of children, is banned in several European countries and several states in the United States.^29^ It is also argued that smoking in vehicles with children could be considered a form of child abuse.^30^ As to SHS at workplace/school, our results suggested that enhancing individual knowledge by itself might not work well to reduce SHS. A complete smoking ban at workplaces, including restaurants and bars, is necessary to eliminate SHS for employees.^19^ It has been reported that a complete smoking ban in restaurants and bars does not lead to revenue loss.^31^^–^^33^

In addition to a population strategy, the concept of proportionate universalism is also needed to reduce social inequalities in SHS.^34^ Proportionate universalism refers to interventions with greater proportionate scale and intensity on more disadvantaged groups.^35^ It has been discussed that population strategies alone do not necessary reduce health inequalities, and the possibility of increment of inequalities has been reported.^36^ For example, though many studies has reported the effectiveness of price/tax increment on smoking cessation and inequalities in smoking,^37^ several studies suggests that price/tax inclement has a larger effect on people with higher education than those with lower education.^37^ In Japan, a decline in the smoking rate was observed for all socioeconomic positions when the price of cigarettes increased in 2010; however, the reduction was not larger among people with lower SES comparing to those with higher SES.^38^

In Japan, cigarettes are available at an affordable price, and over 30% of adults were exposed to SHS in 2013.^25^ The prevalence of SHS in Japan is similar to the worldwide average.^1^ However, unfortunately, Japan has yet to introduce a complete smoking ban law, despite the fact that Japan has approved The World Health Organization Framework Convention on Tobacco Control, which recommends complete smoking bans.^39^ In 2015, the Industrial Safety and Health Act, a Japanese law for work environments, was amended and it became the employer’s responsibility to ban SHS in the workplace; however, the law does not mandate complete smoking bans, and partial smoking bans are applicable at workplaces and restaurants.^40^ Partial smoking ban is less effective to reduce SHS than complete smoking ban.^41^ Introducing smoking restriction policy without penalties would depend on companies’ compliance, so it might be likely to be introduced in higher occupational grades.^19^^,^^41^ More comprehensive, strict smoking restriction laws that include public places are needed to reduce the negative health effects of smoking.

This study is rare because inequalities in SHS are scarcely reported in Japan.^19^ We found that the associations between knowledge of tobacco’s adverse effect on health and SHS are different between SHS at home and that at workplace/school. The study participants were randomly selected from all adults in one area of Japan, and the response rate was quite high (92.8%). Since people with low SES are generally not likely to respond to surveys, a high response rate is important to evaluate actual social gradients. On the other hand, the present study also has several limitations. First, this was a cross-sectional study, so causal inference of the association between SES and SHS is limited. However, it is unreasonable that being exposed to SHS in adulthood causes low educational attainment or low income. Therefore, we believe that reverse causation is not likely to exist in this study. Second, all information was self-reported. However, self-reported exposure to SHS is well correlated with biological SHS measurements.^42^ Third, this study targeted only one of the 47 prefectures in Japan; therefore, generalizability of the present study is limited. However, the prevalence of active smoking in this survey was similar to that of a national survey. Therefore, we believe that this study could be extrapolated to all of Japan.

SHS should be one of the targets to reduce health inequality. Social inequalities are a central concern of the cigarette epidemic.^43^^,^^44^ The inequality in smoking-related diseases may be partly caused by inequalities in SHS. This study investigated social inequalities in secondhand smoke (SHS) in Japan, a country without national smoking restriction legislation with penalties for smoking in public places, and highlighted the importance of policies changing smoker-friendly environments.

## References

[1] Oberg M, Jaakkola MS, Woodward A, Peruga A, Prüss-Ustün A Worldwide burden of disease from exposure to second-hand smoke: a retrospective analysis of data from 192 countries. Lancet. 2011;377(9760):139–146. 10.1016/S0140-6736(10)61388-8 21112082

[2] Eriksen M, Mackay J, Schluger N, Gomeshtapeh FI, Drope J. *The Tobacco Atlas*. Fifth. Atlanta: American Cancer Society; 2015.

[3] United States Department of Health and Human Services. The Health Consequences of Involuntary Exposure to Tobacco Smoke. 2006. http://www.ncbi.nlm.nih.gov/books/NBK44324/. Accessed April 26, 2016.

[4] Max W, Sung HY, Shi Y Deaths from secondhand smoke exposure in the United States: economic implications. Am J Public Health. 2012;102(11):2173–2180. 10.2105/AJPH.2012.300805 22994180 PMC3477960

[5] Ong MK, Glantz SA Cardiovascular health and economic effects of smoke-free workplaces. Am J Med. 2004;117(1):32–38. 10.1016/j.amjmed.2004.02.029 15210386

[6] Parrott S, Godfrey C Economics of smoking cessation. BMJ. 2004;328(7445):947–949. 10.1136/bmj.328.7445.947 15087348 PMC390220

[7] Yao T, Sung HY, Mao Z, Hu TW, Max W The healthcare costs of secondhand smoke exposure in rural China. Tob Control. 2015;24(e3):e221–e226. 10.1136/tobaccocontrol-2014-051621 25335898 PMC4405484

[8] Gartner CE, Hall WD Is the socioeconomic gap in childhood exposure to secondhand smoke widening or narrowing? Tob Control. 2013;22(5):344–348. 10.1136/tobaccocontrol-2011-050297 22467710

[9] Filippidis FT, Agaku IT, Girvalaki C, Relationship of secondhand smoke exposure with sociodemographic factors and smoke-free legislation in the European Union. Eur J Public Health. 2016;26(2):344–349. 10.1093/eurpub/ckv204 26511601

[10] Pisinger C, Hammer-Helmich L, Andreasen AH, Jørgensen T, Glümer C Social disparities in children’s exposure to second hand smoke at home: a repeated cross-sectional survey. Environ Health. 2012;11:65. 10.1186/1476-069X-11-65 22984822 PMC3544183

[11] Ng M, Freeman MK, Fleming TD, Smoking prevalence and cigarette consumption in 187 countries, 1980–2012. JAMA. 2014;311(2):183–192. 10.1001/jama.2013.284692 24399557

[12] Yao T, Sung HY, Mao Z, Hu TW, Max W Secondhand smoke exposure at home in rural China. Cancer Causes Control. 2012;23(Suppl 1):109–115. 10.1007/s10552-012-9900-6 22327886 PMC3637665

[13] McGhee SM, Hedley AJ, Ho LM Passive smoking and its impact on employers and employees in Hong Kong. Occup Environ Med. 2002;59(12):842–846. 10.1136/oem.59.12.842 12468752 PMC1763597

[14] Tsai YWW, Chang LCC, Sung HYY, Hu TWW, Chiou STT The impact of smoke-free legislation on reducing exposure to secondhand smoke: differences across gender and socioeconomic groups. Tob Control. 2015;24(1):62–69. 10.1136/tobaccocontrol-2013-051004 24014636

[15] Abdullah AS, Driezen P, Sansone G, Correlates of exposure to secondhand smoke (SHS) at home among non-smoking adults in Bangladesh: findings from the ITC Bangladesh survey. BMC Pulm Med. 2014;14(1):117. 10.1186/1471-2466-14-117 25027238 PMC4107590

[16] Fischer F, Minnwegen M, Kaneider U, Kraemer A, Khan MM Prevalence and determinants of secondhand smoke exposure among women in Bangladesh, 2011. Nicotine Tob Res. 2015;17(1):58–65. 10.1093/ntr/ntu129 25125322 PMC4832965

[17] Jin Y, Wang L, Lu B, Ferketich AK Secondhand smoke exposure, indoor smoking bans and smoking-related knowledge in China. Int J Environ Res Public Health. 2014;11(12):12835–12847. 10.3390/ijerph111212835 25514143 PMC4276649

[18] Park S, Lim S, Kim J, Lee H, June KJ Socioeconomic disparities in household secondhand smoke exposure among non-smoking adolescents in the Republic of Korea. Glob Public Health. 2017;12(9):1104–1121. 10.1080/17441692.2015.1117119 26654579

[19] Tabuchi T, Nakamura M Disparity of secondhand smoke exposure at home and/or workplace according to age, education and medical insurance in Japan. JACR Monogr. 2014;20:39–48 [in Japanese].

[20] World Health Organization. *WHO Report on the Global Tobacco Epidemic, 2015* World Health Organization; 2015. http://www.who.int/tobacco/global_report/2015/en/. Accessed June 9, 2016.

[21] Solar O, Irwin A. A conceptual framework for action on the social determinants of health. *Soc Determ Heal Discuss Pap 2* *(Policy Pract)*. 2011;(April):79. ISBN 978 92 4 150085 2.

[22] Brant R Assessing proportionality in the proportional odds model for ordinal logistic regression. Biometrics. 1990;46(4):1171–1178. 10.2307/2532457 2085632

[23] Rubin DB. *Multiple Imputation for Nonresponse in Surveys*. Hoboken: John Wiley & Sons; 1987.

[24] Carpenter J, Kenward M. *Multiple Imputation and Its Application*. 1st ed. Hoboken: John Wiley & Sons; 2012.

[25] Ministry of Health, Labour and Welfare. National Health and Nutrition Survey. 2013.

[26] Nazar GP, Lee JT, Arora M, Millett C Socioeconomic inequalities in secondhand smoke exposure at home and at work in 15 low- and middle-income countries. Nicotine Tob Res. 2016;18(5):1230–1239. 10.1093/ntr/ntv261 26610936 PMC4826490

[27] Brown T, Platt S, Amos A Equity impact of population-level interventions and policies to reduce smoking in adults: a systematic review. Drug Alcohol Depend. 2014;138:7–16. 10.1016/j.drugalcdep.2014.03.001 24674707

[28] Pizacani BA, Martin DP, Stark MJ, Koepsell TD, Thompson B, Diehr P Household smoking bans: which households have them and do they work? Prev Med. 2003;36(1):99–107. http://www.ncbi.nlm.nih.gov/pubmed/12473430. Accessed February 22, 2017. 12473430 10.1006/pmed.2002.1123

[29] Black LA. Smoking in private vehicles carrying children. *North Irel Assem Res Inf Serv Res Pap*. 2016;27/16.

[30] Goldstein AO Is exposure to secondhand smoke child abuse? Yes. Ann Fam Med. 2015;13(2):103–104. 10.1370/afm.1764 25755029 PMC4369587

[31] Cornelsen L, McGowan Y, Currie-Murphy LM, Normand C Systematic review and meta-analysis of the economic impact of smoking bans in restaurants and bars. Addiction. 2014;109(5):720–727. 10.1111/add.12486 24529192

[32] López CMG, Ruiz JAJ, Shigematsu LMR, Waters HR The economic impact of Mexico City’s smoke-free law. Tob Control. 2011;20(4):273–278. 10.1136/tc.2010.036467 21292808 PMC3122880

[33] Glantz SA, Charlesworth A Tourism and hotel revenues before and after passage of smoke-free restaurant ordinances. JAMA. 1999;281(20):1911–1918. 10.1001/jama.281.20.1911 10349895

[34] Kipping RR, Smith M, Heron J, Hickman M, Campbell R Multiple risk behaviour in adolescence and socio-economic status: findings from a UK birth cohort. Eur J Public Health. 2015;25(1):44–49. 10.1093/eurpub/cku078 24963150 PMC4304374

[35] Review TM, Marmot M, Atkinson T, et al. Fair Society, Healthy Lives: The Marmot Review, strategic review of health inequalities in England post—2010. *M Marmot*. 2010. doi:10.1136/bmj.c1191. 10.1136/bmj.c1191

[36] Lorenc T, Petticrew M, Welch V, Tugwell P What types of interventions generate inequalities? Evidence from systematic reviews. J Epidemiol Community Health. 2013;67(2):190–193. 10.1136/jech-2012-201257 22875078

[37] Thomas S, Fayter D, Misso K, Population tobacco control interventions and their effects on social inequalities in smoking: systematic review. Tob Control. 2008;17(4):230–237. 10.1136/tc.2007.023911 18426867 PMC2565568

[38] Tabuchi T, Nakamura M, Nakayama T, Miyashiro I, Mori J, Tsukuma H Tobacco price increase and smoking cessation in Japan, a developed country with affordable tobacco: a national population-based observational study. J Epidemiol. 2016;26(1):14–21. 10.2188/jea.JE20140183 26277880 PMC4690736

[39] World Health Organization. MPOWER. 2015. http://www.who.int/tobacco/mpower/en/. Accessed April 26, 2016.

[40] Ministry of Health, Labour and Welfare. Industrial Safety and Health Act. 2015. http://www.mhlw.go.jp/file/06-Seisakujouhou-11200000-Roudoukijunkyoku/T150519K0020.pdf.

[41] Tabuchi T, Colwell B Disparity and Trends in Secondhand Smoke Exposure among Japanese Employees, Particularly Smokers vs Non-Smokers. PLoS One. 2016;11(4):e0152096. 10.1371/journal.pone.0152096 27050819 PMC4822844

[42] Brunekreef B, Leaderer BP, van Strien R, Using nicotine measurements and parental reports to assess indoor air: the PIAMA birth cohort study. Prevention and Incidence of Asthma and Mite Allergy. Epidemiology. 2000;11(3):350–352. 10.1097/00001648-200005000-00023 10784258

[43] Lopez AD, Collishaw NE, Piha T A descriptive model of the cigarette epidemic in developed countries. Tob Control. 1994;3(3):242 10.1136/tc.3.3.242

[44] Graham H Why social disparities matter for tobacco-control policy. Am J Prev Med. 2009;37(2)(Suppl):S183–S184. 10.1016/j.amepre.2009.05.007 19591761

